# Influence of Early Feeding Practices on Oral Microbiota Composition During Infancy and Potential Implications for Early Childhood Caries: A Systematic Review

**DOI:** 10.3390/nu18132138

**Published:** 2026-07-02

**Authors:** Marta Ibor-Miguel, Davinia Pérez-Sánchez, Laura Marques-Martínez, Juan Ignacio Aura-Tormos, Clara Guinot-Barona, Esther García Miralles

**Affiliations:** 1 Dentistry Department, Faculty of Medicine and Health Sciences, Catholic University of Valencia San Vicente Mártir, 46001 Valencia, Spain; marta.ibor@ucv.es (M.I.-M.); davinia.perez@ucv.es (D.P.-S.); laura.marques@ucv.es (L.M.-M.); 2 Doctoral School, Catholic University of Valencia San Vicente Mártir, 46001 Valencia, Spain; 3 Department of Stomatology, Faculty of Medicine and Dentistry, University of Valencia, 46010 Valencia, Spain; juan.aura@uv.es (J.I.A.-T.); m.esther.garcia@uv.es (E.G.M.)

**Keywords:** oral microbiota, early feeding practices, breastfeeding, infant formula, early childhood caries, ECC, infancy, 16S rRNA, systematic review, PRISMA

## Abstract

Background: Early feeding practices are among the most influential determinants of the infant oral microbiota during the first years of life. Breastfeeding provides bioactive components—immunoglobulins, human milk oligosaccharides (HMOs), and commensal bacteria—that may shape microbial colonisation patterns with long-term implications for oral health. However, the nature, magnitude, and clinical relevance of these effects remain poorly characterised, particularly with regard to early childhood caries (ECC) risk. Objectives: The primary objective was to evaluate the association between early feeding practices and oral microbiota composition during infancy. A secondary exploratory objective was to assess whether feeding-associated microbiota differences had been linked to subsequent dental caries outcomes. Methods: A systematic review was conducted in accordance with PRISMA 2020 guidelines. PubMed, Scopus, Web of Science, and Embase were searched from January 2010 to June 2026. Eligible studies compared at least two feeding groups and measured oral microbiota directly using culture-independent methods (16S rRNA gene sequencing, metagenomics, or quantitative PCR targeting multiple taxa). Study selection, data extraction, and risk of bias assessment using the ROBINS-E tool were performed independently. Qualitative synthesis was conducted given clinical and methodological heterogeneity. Results: Of 8582 records identified, 12 studies met the inclusion criteria (sample size range: 12–448 participants; age range at microbiota assessment: 2 days–14 years, although eligibility was based on feeding exposure during infancy; six countries). Most included studies reported differences in oral microbiota composition associated with feeding type. During the first months of life, breastfed infants generally showed lower oral microbial diversity and higher abundance of *Lactobacillus*, the *Streptococcus mitis* group and *Bifidobacterium* compared with formula-fed infants, who exhibited greater alpha diversity, higher transmission of maternal oral bacteria, and higher abundance of *Prevotella* and *Actinomyces*. Effects were most pronounced in the first three months of life and attenuated by 12 months in most cohorts. Only one study reported subsequent dental caries outcomes after early-life microbiota assessment, finding that *Streptococcus cristatus* abundance at three months was associated with dental caries at nine years of age, and that longer breastfeeding duration (≥12 months) was associated with a distinct microbiota profile and lower caries rates in this single available longitudinal study. Risk of bias was low in two studies, moderate in six, and high in four. Publication bias could not be formally evaluated. Conclusions: Early feeding practices are associated with measurable differences in oral microbiota composition during infancy, particularly during the first months of life. However, evidence linking these microbiota differences to subsequent dental caries outcomes remains extremely limited, with only one included study assessing later caries development. Therefore, the clinical significance of feeding-associated microbiota profiles remains uncertain and should be investigated through well-designed prospective longitudinal studies.

## 1. Introduction

Early childhood caries (ECC) is defined as the presence of one or more decayed, missing or filled tooth surfaces in children younger than 71 months and constitutes the most prevalent chronic infectious disease of childhood globally [1]. Its prevalence ranges from approximately 10% to 60% in high-income countries and may exceed 80% in low- and middle-income settings, disproportionately affecting the most socioeconomically vulnerable populations [2]. Beyond its oral consequences, ECC is associated with pain, nutritional compromise, impaired growth, sleep disruption and reduced quality of life, with downstream effects on school performance and socioemotional development [3].

The aetiology of ECC is multifactorial, involving the interaction of fermentable dietary carbohydrates, susceptible tooth surfaces, time and a dysbiotic oral microbial community [4]. *Streptococcus mutans* has historically been regarded as the primary cariogenic pathogen; however, evidence from 16S rRNA gene sequencing and metagenomic studies has shown that ECC is associated with a broader polymicrobial dysbiosis involving *Streptococcus mutans*, *Veillonella* spp., *Prevotella* spp., *Actinomyces* spp., *Lactobacillus* spp. and *Candida albicans*, among others [5,6]. Understanding the factors that shape oral microbiota composition during early infancy—the period of initial microbial colonisation and ecological succession—is therefore of critical importance for ECC prevention [7].

Feeding practices during the first months of life represent one of the most modifiable determinants of early oral microbiota development. The World Health Organisation recommends exclusive breastfeeding for the first six months of life, followed by continued breastfeeding alongside complementary foods until at least two years of age [8]. Human milk is not a sterile fluid; it contains a diverse microbiota, commonly including *Streptococcus*, *Staphylococcus* and *Lactobacillus* spp., as well as human milk oligosaccharides that act as selective prebiotics for beneficial commensals, immunomodulatory proteins such as secretory IgA, lactoferrin and lysozyme, and antimicrobial enzymes that collectively shape early microbial colonisation [9,10]. Infant formula, by contrast, represents a different nutritional and biological matrix. Although modern formulas may include added prebiotics or bioactive compounds, they do not fully reproduce the complexity, variability or host-adapted immunological functions of human milk, and their distinct fermentable carbohydrate content may plausibly favour different patterns of oral microbial acquisition and ecological succession during infancy [11].

Despite a growing body of culture-independent microbiome research, the relationship between early feeding practices, oral microbiota composition and later caries risk remains insufficiently characterised. Previous systematic reviews and meta-analyses have examined the association between breastfeeding and dental caries, but their findings have been inconsistent, partly because breastfeeding duration, nocturnal feeding, dietary sugar intake, fluoride exposure, oral hygiene practices and socioeconomic factors are difficult to disentangle [12,13]. More importantly, those reviews did not focus on oral microbiota composition as a potential biological mediator between feeding exposure and caries development. A systematic review centred on this microbiological pathway is therefore warranted.

The present systematic review aimed to synthesise the available evidence on whether early feeding practices, particularly exclusive or predominant breastfeeding compared with infant formula or mixed feeding, are associated with differences in oral microbiota composition in children aged 0–71 months. Secondary objectives were to identify microbial taxa and diversity indices consistently associated with feeding type, and to determine whether feeding-associated microbiota differences have been linked to subsequent early childhood caries or later dental caries outcomes.

## 2. Methods

**Figure 1:**
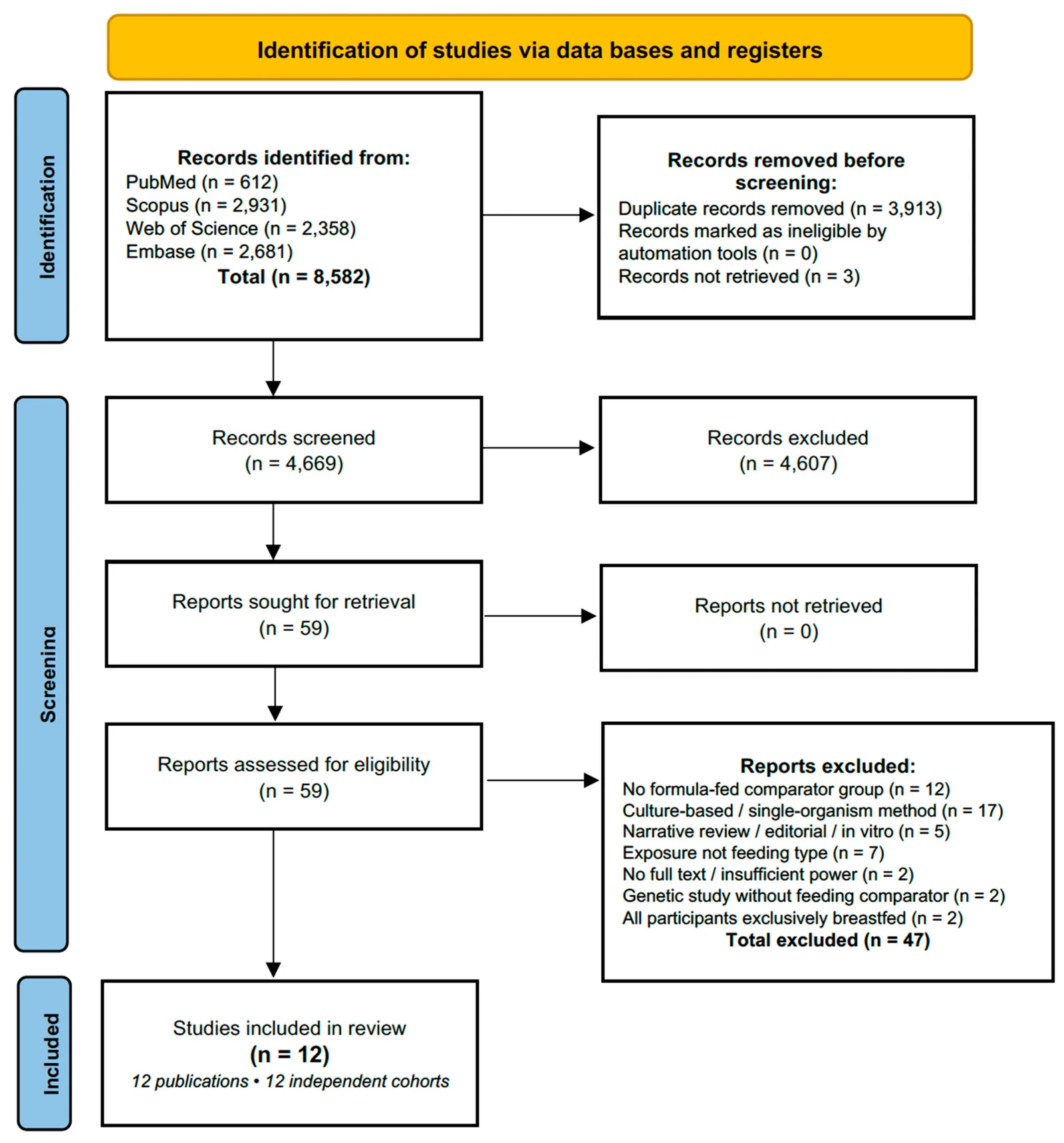
PRISMA 2020 flow diagram of study selection process. ECC (early childhood caries); BF (breastfed); FF (formula-fed).

This systematic review was conducted and reported in accordance with the Preferred Reporting Items for Systematic Reviews and Meta-Analyses (PRISMAs) 2020 guidelines [14]. The PRISMA 2020 flow diagram is included as Figure 1 in the main text. This systematic review was prospectively registered in PROSPERO prior to submission (registration number CRD420261419799).

### 2.1. Eligibility Criteria

Studies were considered eligible when they included children aged 0–71 months at the time of the initial oral microbiota assessment or at the time of feeding exposure. For longitudinal cohort studies with follow-up extending beyond 71 months, eligibility was determined according to the child’s age at the first microbiota measurement. No restrictions were applied regarding sex, gestational age, or country of origin. One study with retrospective exposure ascertainment, in which oral microbiota was measured at 10–14 years of age, was retained as a sensitivity study because it provided a unique long-term perspective; however, its findings were interpreted separately throughout the synthesis [15]. The exposure of interest was exclusive or predominant breastfeeding as the primary feeding modality, while the comparator was infant formula feeding, either exclusive or combined with breastfeeding. The primary outcome was oral microbiota composition assessed using culture-independent methods, including 16S rRNA gene sequencing, metagenomics, or multiplexed quantitative PCR targeting three or more taxa. Culture-based studies limited to the quantification of one or two specific organisms were excluded. The secondary outcome was clinically assessed dental caries, including ECC when assessed before 71 months of age, or later dental caries outcomes in longitudinal studies in which feeding exposure or oral microbiota had been assessed during infancy. Eligible study designs included prospective and retrospective cohort studies, case–control studies, and cross-sectional studies published between January 2010 and June 2026.

Studies were excluded if they were animal or in vitro investigations, if feeding type was not the primary exposure of interest, or if feeding was only included as a covariate. This criterion was adopted to ensure that feeding modality constituted the principal exposure of interest and to minimise interpretative ambiguity arising from studies in which feeding was only included as a secondary covariate. Studies exclusively reporting culture-based quantification of a single cariogenic organism, such as *Streptococcus mutans* alone on MSB agar, were also excluded. Editorials, narrative reviews, conference abstracts without primary data, studies without accessible full text, and studies in which more than 90% of the sample was older than 71 months at the time of microbiota measurement were not considered eligible.

### 2.2. Information Sources and Search Strategy

A comprehensive electronic search was conducted on 6 June 2026 in the following four databases: PubMed/MEDLINE, Scopus, Web of Science Core Collection, and Embase. The search combined terms related to the following three concepts: (1) early feeding practices (breastfeeding, breast milk, formula feeding, infant formula, mixed feeding, and bottle feeding); (2) oral microbiota (oral microbiome, oral microbiota, 16S rRNA, saliva microbiome, dental plaque microbiome, oral bacteria, and oral microbial diversity); and (3) early childhood caries and related outcomes (early childhood caries, ECC, dental caries, *Streptococcus mutans*, dental biofilm, and cariogenic bacteria). Full search strings for each database are provided in Supplementary Table S1. No language restrictions were applied in PubMed/MEDLINE, Web of Science or Embase. In Scopus, records were limited to English- and Spanish-language publications for practical screening purposes. All searches were restricted to publications from January 2010 to June 2026.

### 2.3. Screening and Eligibility Assessment

Records were exported to a deduplicated RIS file and screened in two phases. In Phase 1, two independent reviewers screened titles and abstracts against the eligibility criteria. In Phase 2, full texts of potentially eligible records were retrieved and assessed. Disagreements were resolved by discussion and, where necessary, by consultation with a third reviewer.

### 2.4. Data Extraction

Data were extracted independently by one reviewer and verified by a second using a pre-specified extraction form (Supplementary Table S2). The following variables were extracted: study identification (authors, year, country, journal, and DOI); study design; participant characteristics (sample size, age, and inclusion/exclusion criteria); feeding type definitions and measurement method; microbiological method (16S rRNA gene region, sequencing platform, reference database, and bioinformatic pipeline); biological sample type and sampling age; diversity indices (alpha: Shannon, observed OTUs/ASVs, and Faith’s PD; beta: Bray–Curtis, UniFrac); differential abundance results for specific taxa; and dental caries outcomes, including diagnostic index, prevalence, dmft/dmfs values, and reported statistical associations.

### 2.5. Risk of Bias Assessment

Risk of bias was assessed using the ROBINS-E (Risk Of Bias In Non-randomised Studies of Exposures) tool [16], which evaluates the following seven domains: (D1) confounding; (D2) selection of participants; (D3) classification of exposure; (D4) deviations from intended exposures; (D5) missing data; (D6) measurement of outcomes; and (D7) selection of the reported result. The one randomised controlled trial identified included a non-randomised breastfeeding reference group; ROBINS-E was therefore applied to the breastfeeding comparison within that study [17]. For each domain, studies were rated as low, moderate, high, or critical risk of bias, with an overall judgement assigned according to ROBINS-E guidance. Domain D4 was not applicable to observational feeding studies.

### 2.6. Synthesis

Quantitative meta-analysis was not performed owing to substantial heterogeneity in study designs, age ranges, feeding definitions, microbiological methods, sequencing platforms, and reference databases. A narrative synthesis was conducted, organised by (i) diversity indices (alpha and beta diversity), (ii) specific taxa consistently associated with feeding type, and (iii) associations with dental caries outcomes. Consistency of direction of effects across studies was assessed qualitatively. Subgroup patterns were described for age at measurement, feeding definition, and sequencing platform.

## 3. Results

### 3.1. Study Selection

The electronic database search yielded 8582 records (PubMed: 612; Scopus: 2931; Web of Science: 2358; and Embase: 2681). After removal of 3913 duplicates, 4669 unique records were screened at the title and abstract stage. Of these, 4610 were excluded, and 59 full-text articles were assessed for eligibility. Forty-seven articles were excluded at the full-text stage for the following reasons: no formula-fed comparator group (*n* = 12), oral microbiota measurement based only on selective culture for a single organism (*n* = 17), narrative review, editorial or in vitro design (*n* = 5), feeding type not the primary exposure (*n* = 7), full text unavailable (*n* = 2), genetic study without a feeding comparator (*n* = 1), exclusively breastfed sample (*n* = 2), or duplicate/non-independent cohort report without additional eligible data for the present synthesis (*n* = 1). Twelve studies met all inclusion criteria and were included in the synthesis [15,17,18,19,20,21,22,23,24,25,26,27]. The selection process is depicted in Figure 1. An albatross plot of alpha-diversity results across included studies is provided in Supplementary Figure S1.

### 3.2. Characteristics of Included Studies

Table 1 summarises the characteristics of the 12 included studies [15,17,18,19,20,21,22,23,24,25,26,27]. Studies were published between 2013 and 2022, conducted in six countries (Australia, Finland, Japan, Spain, Sweden and the USA), and included a total of 1747 mother–infant pairs or infants. Sample sizes ranged from 12 participants in the study by Oba et al. [21] to 448 participants in the study by Kageyama et al. [25]. Most studies used cross-sectional (*n* = 6) or prospective cohort designs (*n* = 5), while one was a randomised controlled trial that included a non-randomised breastfeeding reference group [17]. Age at microbiota measurement ranged from two days to 14 years; however, 10 of the 12 studies measured oral microbiota within the first 24 months of life. The most commonly used microbiological approach was 16S rRNA V3–V4 region Illumina MiSeq sequencing, although sequencing regions, platforms and bioinformatic pipelines varied across studies. One study used full-length 16S rRNA sequencing with PacBio Sequel II [25], and one used the Human Oral Microbiome Identification Microarray combined with culture-based methods and quantitative PCR [20]. Only one study reported subsequent dental caries outcomes after early-life microbiota assessment [18].

### 3.3. Risk of Bias

**Figure 2:**
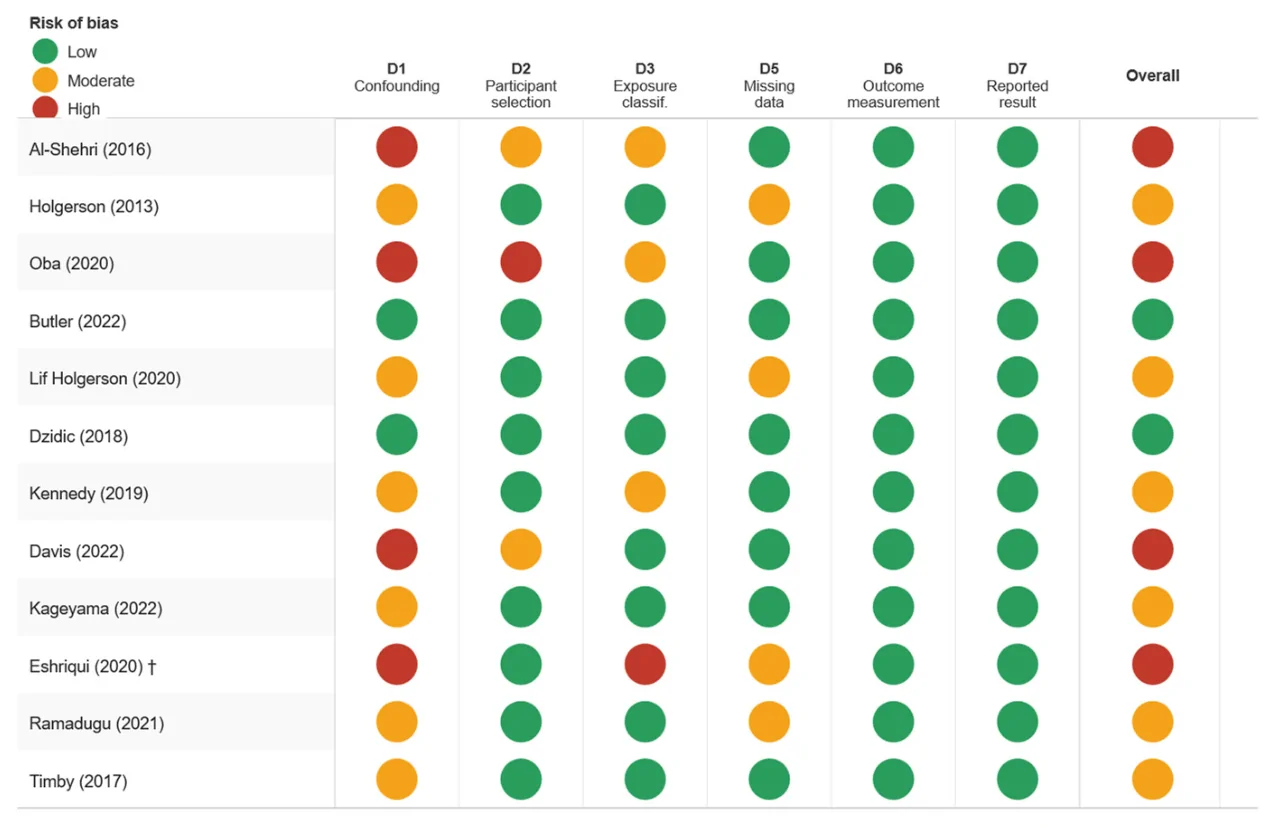
Risk of bias assessment of included studies using the ROBINS-E tool. D1, confounding; D2, selection of participants; D3, classification of exposure; D5, missing data; D6, measurement of outcomes; D7, selection of the reported result. Domain D4 (deviations from intended exposures) was not applicable. † assessed microbiota composition 10–14 years after the feeding exposure period; this study was retained as a sensitivity analysis. Green, low risk of bias; amber, moderate risk of bias; red, high risk of bias [15,17,18,19,20,21,22,23,24,25,26,27].

Risk of bias was assessed as low in two studies [18,22], moderate in six [17,20,23,25,26,27], and high in four [15,19,21,24] (Figure 2). For Kennedy et al. [27], risk of bias was rated as moderate; the authors explicitly acknowledged that the breastfeeding effect was likely attenuated because the first saliva sampling occurred at six months of age, after most infants had already been introduced to complementary foods, and the pre-specified response category for exclusive breastfeeding duration was capped at ≥5 months regardless of subsequent solid food introduction. The most common sources of bias were inadequate control for confounding and retrospective or self-reported exposure classification. The study by Eshriqui et al. [15] was unique in measuring oral microbiota in adolescents 10–14 years after the feeding exposure. Although its eligibility was based on the age at feeding exposure during infancy, the large temporal gap between exposure and microbiota measurement introduces substantial confounding from intervening dietary, hygienic and microbiological exposures. Accordingly, its findings were interpreted as a sensitivity perspective rather than as a primary result throughout this review.

### 3.4. Effect of Feeding Practices on Oral Microbiota Alpha Diversity

Alpha diversity was reported by 11 of the 12 included studies. A broadly consistent pattern emerged during early infancy as follows: formula-fed infants tended to exhibit greater oral microbial species richness and/or evenness than exclusively breastfed infants during the first six months of life. This finding was reported across several studies using different sampling and sequencing approaches [17,19,20,21,22,23,24,25,26]. The magnitude of this difference was most pronounced in the study by Kageyama et al. [25], in which formula-fed infants at four months had a mean of 43.9 ± 17.7 ASVs compared with 27.3 ± 11.3 ASVs in breastfed infants (*p* < 0.001). Ramadugu et al. [26] similarly found lower Shannon diversity in exclusively breastfed infants at two months than in formula-fed infants (2.77 vs. 3.12, *p* = 0.003), although this difference was not sustained at 12 months.

The temporal dynamics of these differences were examined in four longitudinal studies. Three of these studies reported convergence of alpha diversity between feeding groups by 8–12 months of age, suggesting that the influence of breastfeeding on microbial richness is primarily a phenomenon of early infancy [22,23,26]. In contrast, Dzidic et al. [18] found that breastfeeding duration of ≥12 months was associated with significantly greater oral microbial diversity at 24 months and seven years of age compared with breastfeeding for <6 months (*p* < 0.05), suggesting that duration, rather than the mere presence of breastfeeding, may determine whether diversity effects persist.

Eshriqui et al. [15], uniquely assessing adolescents aged 10–14 years, found no differences in overall alpha diversity, measured using Shannon or Inverse Simpson indices, between participants who had or had not received infant formula in their first six months of life. This absence of effect is consistent with either a genuine lack of long-term alpha diversity differences or attenuation by the many intervening exposures over the decade separating feeding practice from microbiota measurement. This study is retained as a sensitivity analysis given the long interval between feeding exposure and microbiota measurement (see Sensitivity Analysis: Long-Term Follow-Up Study below).

### 3.5. Effect of Feeding Practices on Oral Microbiota Beta Diversity

Ten studies reported beta diversity analyses. All studies that performed statistical testing found significant differences in overall oral microbiota composition between breastfed and formula-fed or mixed-fed infants. Methods included Bray–Curtis dissimilarity with PERMANOVA, principal coordinate analysis and partial least squares regression [17,18,19,20,21,22,23,24,25,26,27].

The most robust longitudinal evidence came from Dzidic et al. [18], who demonstrated that the oral microbiota of children with longer breastfeeding duration (≥12 months) diverged from that of shorter-breastfed children by 24 months of age and remained compositionally distinct at seven years (PERMANOVA *p* = 0.002). Similarly, Timby et al. [17] found that the breastfeeding reference group clustered separately from formula-fed infants in principal coordinate analysis space at four months, with this separation diminished but still detectable at 12 months. Lif Holgerson et al. [23] found the most pronounced beta diversity differences between exclusive breastfeeding and formula feeding at three months, with microbiota in mixed-fed infants occupying an intermediate position. By contrast, Eshriqui et al. [15] found no significant difference in beta diversity in adolescence associated with early feeding type (*p* = 0.881), consistent with their null alpha diversity findings.

### 3.6. Specific Taxa Associated with Feeding Type

#### 3.6.1. Taxa Enriched in Breastfed Infants

Across the included studies, several microbial taxa were reported at higher abundance in breastfed infants than in formula-fed or mixed-fed infants. *Lactobacillus* spp. were detected in 27–32% of breastfed infants at three months of age, whereas they were not detected in exclusively formula-fed infants in the study by Holgerson et al. [20]. Timby et al. [17] reported a similar pattern, with higher detection of *Lactobacillus gasseri* and *Lactobacillus rhamnosus* in the breastfeeding reference group according to partial least squares regression. This finding is biologically plausible, given the documented presence of *Lactobacillus* spp. in human milk [9,10]. Streptococcal taxa also appeared to differ according to feeding modality. Butler et al. [22] found significantly higher abundance of the *Streptococcus mitis* group in breastfed infants at two months of age, while Davis et al. [24] reported greater abundance of *Streptococcus* more broadly in exclusively breastfed infants than in mixed-fed infants at six weeks. In addition, *Bifidobacterium* spp., particularly *Bifidobacterium breve*, were characteristic of the breastfeeding reference profile described by Timby et al. [17], with higher relative abundance than in formula-fed groups at both four and 12 months of age. Evidence for longer-term microbial signatures was more limited, but Eshriqui et al. [15] found higher abundance of *Eubacterium* and *Veillonella* operational taxonomic units in adolescents who had not received formula during infancy, suggesting a possible legacy effect on selected taxa despite the absence of differences in global microbial diversity. Finally, Davis et al. [24] reported higher relative abundance of *Gemella* spp. in exclusively breastfed infants at six weeks compared with mixed-fed infants.

#### 3.6.2. Taxa Enriched in Formula-Fed Infants

Formula-fed infants also showed enrichment of several microbial taxa across the included studies, although the reported patterns varied by age, sampling method and taxonomic resolution. Kageyama et al. [25] found significantly higher abundance of *Prevotella melaninogenica* and *Granulicatella adiacens* in formula-fed infants than in breastfed infants. Both species were interpreted as being predominantly of maternal oral origin, which was consistent with the broader observation that formula-fed infants harboured a significantly greater proportion of maternally derived oral bacteria than breastfed infants. Similar shifts towards anaerobic and early plaque-associated taxa were reported by Oba et al. [21] and Ramadugu et al. [26], who found higher abundance of *Actinomyces* spp. and *Prevotella* spp. among formula-fed or non-breastfed infants. In the Ramadugu et al.’s study [26], relative abundance of *Veillonella* and *Prevotella* was 37.3% and 47.3% lower, respectively, in breastfed infants than in non-breastfed infants after adjustment for age and delivery mode, indirectly supporting their enrichment in the non-breastfed group. At a broader taxonomic level, Al-Shehri et al. [19] reported enrichment of the *Bacteroidetes*/*Prevotella* profile in formula-fed neonates, together with depletion of *Actinobacteria* and *Proteobacteria*. Holgerson et al. [20] also observed higher abundance of *Haemophilus* spp. in formula-fed infants. Finally, Timby et al. [17] reported a higher prevalence of *Streptococcus mutans* in formula-fed infants than in breastfed infants at 12 months of age, although this difference did not remain statistically significant after correction for multiple testing.

#### 3.6.3. Taxa Associated with Caries Risk

Only one study provided data linking early-life oral microbiota profiles to subsequent dental caries outcomes [18]. In this prospective cohort of 90 children followed from three months to nine years of age, *Streptococcus cristatus* abundance at three months was significantly associated with subsequent caries development at nine years, being higher in children who later developed caries than in caries-free children (*p* = 0.026). Children breastfed for ≥12 months had a more diverse oral microbiota at 24 months and seven years and a lower caries rate at nine years compared with those breastfed for <6 months, although the statistical relationship between microbiota composition and caries was not formally modelled with feeding as a mediator.

The remaining 11 included studies did not assess dental caries as an outcome, and therefore the clinical significance of the microbiota differences described above, in terms of caries risk, cannot be directly inferred from the current evidence base.

### 3.7. Temporal Dynamics and Effect Modification

Temporal analysis across longitudinal studies revealed the following consistent pattern: feeding-associated microbiota differences were most pronounced in the first three months of life, partially attenuated between three and 12 months, and largely disappeared by 12–24 months in most comparisons [22,23,26]. However, when breastfeeding was prolonged beyond 12 months, diversity and compositional effects appeared to persist into school age [18]. This temporal gradient is consistent with the increasing influence of environmental exposures, including teething, complementary foods and social contacts, which may progressively override the early feeding signal.

Notable effect modification was observed by breastfeeding duration. Studies classifying exposure according to prolonged breastfeeding, particularly ≥12 months, found more robust and persistent effects than those using a binary breastfeeding yes/no classification at a single time point [18,23].

## 4. Discussion

This systematic review synthesised evidence from 12 studies examining the relationship between early feeding practices, oral microbiota composition and caries-related outcomes in children [15,17,18,19,20,21,22,23,24,25,26,27]. The principal finding is that breastfeeding is associated with a distinct early oral microbiota, particularly during the first months of life. Compared with formula-fed infants, breastfed infants generally showed lower species richness during early infancy and higher abundance of selected commensal taxa, including *Lactobacillus*, the *Streptococcus mitis* group and *Bifidobacterium* [17,20,22,24]. Formula-fed infants showed greater early microbial diversity and higher abundance of taxa associated with a more complex, maternally derived oral microbiota, including *Prevotella*, *Actinomyces* and *Granulicatella* [19,21,25,26]. These differences were most pronounced during the first three months of life, attenuated by 12 months in most cohorts, and appeared to persist only when breastfeeding was prolonged beyond one year [18,22,23,26]. Direct evidence linking early feeding-associated microbiota differences to later dental caries was confined to a single prospective cohort, in which *Streptococcus cristatus* abundance at three months was associated with subsequent caries development at nine years of age [18].

### 4.1. Biological Plausibility

The observed microbiota differences are biologically plausible. Human milk contains human milk oligosaccharides, which selectively promote the growth of *Bifidobacterium* and *Lactobacillus* spp. in the gastrointestinal tract and may also influence microbial ecology in the oral cavity. It also contains secretory IgA, lactoferrin and xanthine oxidase, which collectively modulate microbial adherence and proliferation [10,28]. The antimicrobial enzyme xanthine oxidase, present in human milk, has been shown to react with xanthine and hypoxanthine in neonatal saliva to generate reactive oxygen species with antibacterial activity, potentially limiting colonisation by maternally derived bacteria [29]. This mechanism is consistent with the finding by Kageyama et al. [25] that the abundance of maternally transmitted oral bacteria was markedly lower in breastfed than in formula-fed infants, suggesting that human milk may act as a biological barrier to vertical transmission of selected maternal oral bacteria, including potentially cariogenic species.

The higher alpha diversity observed in formula-fed infants may reflect a less selective oral environment in the absence of the same concentration and complexity of human milk bioactive components, allowing a broader range of bacteria, including maternally derived anaerobic and plaque-associated taxa, to colonise earlier. Whether this earlier microbial diversification is beneficial or detrimental for long-term oral health remains unclear. Although greater diversity is generally considered a feature of ecological stability in the mature oral microbiome [30], its significance during early infancy, when microbial succession is still developing, cannot be assumed to be equivalent.

### 4.2. Implications for Caries Risk

The central unresolved question is whether the feeding-associated microbiota differences documented in these studies translate into clinically meaningful differences in caries risk. The only study in this review that measured both early-life oral microbiota and later caries outcomes provides circumstantial but important evidence that the microbiota at three months, a period when feeding-associated differences are most pronounced, may contain compositional signals associated with caries development at nine years of age [18]. The identification of *Streptococcus cristatus* as a candidate early microbiological marker is novel and warrants replication. This organism, a member of the *Streptococcus mitis* group, has been associated with dental plaque maturation and may facilitate subsequent colonisation by *S. mutans* [31].

The finding that formula-fed infants harbour higher abundances of *Prevotella*, *Actinomyces* and *Granulicatella*, organisms associated with a more adult-like and ecologically complex oral biofilm, raises the hypothesis that formula feeding may accelerate oral microbiota maturation [21,25,26]. This premature maturation hypothesis parallels findings in the gut microbiome literature, where formula feeding has been associated with an earlier transition towards an adult-like microbiota composition [32]. Whether this accelerated maturation increases, decreases or has no independent effect on later caries risk requires investigation in prospective studies that integrate feeding data, microbiota profiling and clinical caries assessment.

### 4.3. Limitations of the Evidence Base

Several important limitations must be acknowledged. First, although eligibility was based on feeding exposure during infancy (0–71 months), one study measured oral microbiota in adolescence (10–14 years after feeding exposure) and was retained exclusively as a sensitivity analysis; its findings should not be interpreted alongside primary results. Second, only one study reported subsequent dental caries outcomes after early-life microbiota assessment, which prevented direct evaluation of whether feeding-mediated microbiota differences translate into clinically meaningful differences in caries risk [18]. Third, substantial methodological heterogeneity in sequencing platforms, amplified 16S rRNA regions, reference databases, bioinformatic pipelines and feeding definitions precluded quantitative synthesis. Fourth, exposure classification was predominantly based on parental questionnaires without objective validation, and most studies did not account for nocturnal feeding, feeding frequency or the precise age at which formula was introduced. These factors may be important modifiers of both microbiota composition and caries risk. Fifth, the majority of studies did not adequately control for dietary sugar intake and oral hygiene practices, which are powerful independent determinants of both oral microbiota composition and dental caries. Sixth, all but one study had sample sizes below 250 participants, limiting statistical power for subgroup analyses. Finally, risk of bias was high in four studies, mainly due to insufficient control for confounding or retrospective exposure assessment [15,19,21,24]. Additionally, the grey literature, conference proceedings, preprints and trial registries were not systematically searched, which may have introduced publication bias.

#### 4.3.1. Strength of the Evidence

Interpretation of the findings should consider that only two studies were judged to have low risk of bias, whereas most studies were classified as moderate or high risk. Consequently, confidence in observed feeding-associated microbiota differences remains limited and conclusions should be interpreted cautiously.

#### 4.3.2. Potential Confounding Factors

Feeding practices are closely intertwined with several determinants of oral microbiota development, including delivery mode, antibiotic exposure, maternal oral health, socioeconomic status, timing of complementary feeding, sugar exposure, fluoride use, oral hygiene practices and nocturnal feeding habits. Because many included studies did not adequately adjust for these factors, causal interpretation remains limited.

### 4.4. Comparison with Previous Reviews

Previous systematic reviews examining the association between breastfeeding and ECC have yielded inconsistent results. A 2015 meta-analysis by Avila et al. found no significant association between breastfeeding duration shorter than 12 months and ECC risk but reported an increased risk with prolonged breastfeeding beyond 12 months [12]. A subsequent meta-analysis by Tham et al. similarly found a positive association between prolonged breastfeeding and ECC [13]. These reviews, however, did not examine oral microbiota composition as a potential mechanistic pathway. The present review therefore adds a microbiological perspective to this debate, showing that feeding practices are associated with measurable differences in oral microbial diversity, community structure and taxonomic composition during infancy. Importantly, the microbiota findings do not support a simple interpretation in which breastfeeding is inherently protective or formula feeding is inherently harmful. Rather, they suggest that feeding modality shapes early microbial succession, while the downstream clinical meaning of these shifts probably depends on duration of exposure, nocturnal feeding, dietary sugar intake, fluoride exposure, oral hygiene and broader socioeconomic context.

### 4.5. Recommendations for Future Research

The findings of this review indicate several priorities for future research. First, there is a clear need for longitudinal studies that integrate oral microbiota profiling with clinically assessed caries outcomes. Ideally, these studies should collect oral microbiota samples at multiple time points during early infancy, such as 1, 3, 6, 12 and 24 months, and subsequently follow children until at least five years of age to allow robust caries assessment. Future investigations should also apply standardised feeding exposure classifications, clearly distinguishing exclusive breastfeeding, predominant breastfeeding, mixed feeding and formula-only feeding. Where feasible, these categories should be supported by objective validation and should record not only feeding modality, but also duration, frequency and nocturnal feeding patterns. Adequate adjustment for major confounders will be essential, particularly dietary sugar intake, oral hygiene practices, fluoride exposure, socioeconomic status and mode of delivery. Methodologically, the use of higher-resolution sequencing approaches, such as full-length 16S rRNA gene sequencing or shotgun metagenomics, would allow species- and strain-level characterisation of the oral microbiota and improve the identification of specific cariogenic taxa. Finally, future studies should examine the duration–response relationship between breastfeeding and oral microbiota development, as the studies that assessed prolonged breastfeeding, particularly beyond 12 months, suggested more persistent microbial effects [18,23].

## 5. Conclusions

Early feeding practices are associated with measurable differences in oral microbiota composition during infancy, particularly during the first months of life. Exclusive breastfeeding is generally associated with lower oral microbial diversity during the first months of life and enrichment of selected commensal taxa, including *Lactobacillus*, the *Streptococcus mitis* group and *Bifidobacterium*. Formula feeding is associated with greater early oral microbial diversity and higher abundance of maternally transmitted, ecologically complex taxa, including *Prevotella*, *Actinomyces* and *Granulicatella*. These differences are most pronounced during the first three months of life and tend to attenuate substantially by 12 months, although some compositional effects may persist when breastfeeding is prolonged beyond one year.

Direct evidence linking these feeding-associated microbiota differences to subsequent caries risk remains extremely limited. Only one prospective cohort reported later dental caries outcomes after early-life microbiota assessment, finding that specific oral microbiota profiles at three months were associated with caries development at nine years of age, and that longer breastfeeding duration was associated with a distinct microbiota profile and lower caries rates. Therefore, the clinical significance of feeding-associated microbiota profiles remains uncertain. Well-designed longitudinal studies integrating detailed feeding exposure data, repeated oral microbiota profiling and prospective clinical caries assessment are needed to clarify the role of the oral microbiome as a potential biological mediator between early feeding practices and later caries risk.

## Figures and Tables

**Table 1:** Methodological and clinical characteristics of the included studies.

First Author (Year)	Country	Design	N (BF/FF/Mixed)	Microbiological Method	Age at Sampling	Caries Outcome	RoB	Feeding Definition	Timing of Exposure Assessment	Sample Type	Main Limitations
Al-Shehri et al. (2016) [19]	Australia	Cross-sectional	20/10/8	16S Roche 454 (V1–V2); HOMD; buccal swab	4–8 weeks	No	High	BF: exclusive breastfeeding; FF: exclusive formula; Mixed: BF + formula	4–8 weeks (concurrent cross-sectional)	Buccal swab	Small N (38); no confounder adjustment; single time point
Holgerson et al. (2013) [20]	Sweden	Cross-sectional	120/50/37	HOMIM + culture + qPCR; saliva swab	3 months	No	Moderate	BF: exclusive, no formula; FF: exclusive formula; Mixed: BF + formula	3 months (concurrent cross-sectional)	Saliva swab	Partial confounder adjustment; HOMIM less sensitive than sequencing
Oba et al. (2020) [21]	USA	Cross-sectional	4/4/4	16S MiSeq (V4); SILVA; buccal swab	0–6 months	No	High	BF: exclusive; FF: exclusive formula; Mixed: BF + formula	0–6 months (concurrent cross-sectional)	Buccal swab	Very small sample size (*n* = 12); no confounder adjustment
Butler et al. (2022) [22]	Australia	Cohort	20/—/19	16S Ion Torrent (V1–V2); HOMD; buccal swab	2, 8 and 20 months	No	Low	BF: ≥80% feeds as breastmilk; Mixed: any formula supplementation	Longitudinal: 2, 8, 20 months	Buccal swab	No exclusive formula group; moderate sample size
Lif Holgerson et al. (2020) [23]	Sweden	Longitudinal cohort	~80/~40/~40 *	16S MiSeq (V3–V4); SILVA; saliva	2 days–5 years *	No	Moderate	BF: exclusive, no supplements; FF: exclusive formula; Mixed: BF + formula	Longitudinal: 2 days–5 years; feeding differences only at 3 months	Saliva (Salivette)	Later time points underpowered for feeding comparisons
Dzidic et al. (2018) [18]	Spain/Sweden	Prospective cohort	~30/~30/~30 ^†^	16S MiSeq (V3–V4); SILVA; saliva	3 months–7 years; caries at 9 years	Dental caries at 9 years ^‡^	Low	BF ≥ 12 months vs. <6 months vs. 6–12 months duration	Longitudinal: 3 months–7 years; caries at 9 years	Saliva swab	Feeding classified by duration retrospectively; caries beyond ECC age
Kennedy et al. (2019) [27]	Sweden	Cohort	~30/—/~29	16S MiSeq (V3–V4); SILVA v128; saliva	6–24 months	No	Moderate	BF ≥ 5 months vs. 3–4 months vs. 0–2 months exclusive BF duration	Longitudinal: 6, 12, 24 months (first sample post-weaning for many)	Evening saliva	First sample at 6 months, after complementary feeding; BF capped at ≥5 months
Davis et al. (2022) [24]	USA	Cross-sectional	20/0/13	16S MiSeq (V3–V4); SILVA; buccal swab	6 weeks	No	High	BF: exclusively breastmilk; Mixed: breastmilk + formula; no exclusive FF group	6 weeks (concurrent cross-sectional)	Buccal swab	Small N (33); no exclusive FF comparator; no confounder adjustment
Kageyama et al. (2022) [25]	Japan	Cross-sectional	255/60/131	PacBio full-length 16S; eHOMD; tongue swab	4 months	No	Moderate	BF: exclusive; FF: exclusive formula; Mixed: BF + formula	4 months (concurrent cross-sectional)	Tongue swab	Cross-sectional; self-reported feeding; single time point
Eshriqui et al. (2020) [15]	Finland	Retrospective cross-sectional	175/248/—	16S HiSeq1500 (V3–V4); SILVA; saliva	10–14 years	No	High	No formula in first 6 months vs. any formula in first 6 months	Retrospective: feeding 0–6 months; microbiota at 10–14 years	Saliva (Oragene OG-500)	10–14-year gap between exposure and outcome; major residual confounding
Ramadugu et al. (2021) [26]	USA	Prospective cohort	21/37/42	16S HiSeq (V6); CORE; saliva	2–24 months	No	Moderate	BF: exclusive at 2 months; FF: formula only; Mixed: BF + formula at 2 months	Longitudinal: 2, 9/12, 24 months	Saliva (DNA Genotek)	Appalachian cohort (limited generalisability); V6 region lower resolution
Timby et al. (2017) [17]	Sweden	RCT + BFR ^§^	80/80 + 80/—	16S MiSeq (V3–V4); HOMD; multiple oral sites	4 and 12 months	No	Moderate	BFR: exclusively breastfed (non-randomised); FF: standard or MFGM-enriched formula (randomised)	Longitudinal: 4 and 12 months	Buccal, tongue, alveolar mucosa swabs; tooth at 12 months	BFR not randomised; BF vs. FF comparison is observational

BF, breastfed; FF, formula-fed; Mixed, mixed feeding; HOMD, Human Oral Microbiome Database; HOMIM, Human Oral Microbiome Identification Microarray; SILVA, SILVA rRNA database; CORE, CORE oral microbiome database; eHOMD, expanded Human Oral Microbiome Database; RoB, risk of bias; RCT, randomised controlled trial; BFR, breastfeeding reference group. * Feeding-related differences were only analysable at the 3-month time point. ^†^ Breastfeeding classified as <6, 6–12 or ≥12 months duration. ^‡^ Dental caries assessed at 9 years of age. ^§^ The comparison of interest, BFR versus formula-fed groups, is observational rather than randomised.

## Data Availability

All data generated or analysed during this study are included in this published article and its Supplementary Materials. The PROSPERO registration CRD420261419799.

## Supplementary Materials

The following supporting information can be downloaded at https://www.mdpi.com/article/10.3390/nu18132138/s1, Figure S1: Albatross plot of included studies; Table S1: De-tailed electronic search strategies; Table S2: Data extraction of included studies.
