# Supplementary material for: Influence of Early Feeding Practices on Oral Microbiota Composition During Infancy and Potential Implications for Early Childhood Caries: A Systematic Review
Source: Nutrients. 2026 Jul 2;18(13):2138. doi: 10.3390/nu18132138 (PMC13363697; doi:10.3390/nu18132138)
Supplement: Supplementary file 1 [file nutrients-18-02138-s001.zip › nutrients-4398539-supplementary.pdf]

nutrients
MDPI

Systematic Review

Influence of Early Feeding Practices on Oral Microbiota Composition During Infancy and Implications for Early Childhood Caries Risk: A Systematic Review

Supplementary Materials

Table S1

Detailed Electronic Search Strategies

1. PubMed/MEDLINE

Line	Search concept	Search string
#1	Early feeding — breast-feeding	*("breastfeeding"[MeSH Terms] OR "breast feeding"[Title/Abstract] OR "breast-fed"[Title/Abstract] OR "breast milk"[Title/Abstract] OR "human milk"[Title/Abstract] OR "lactation"[Title/Abstract])*
#2	Early feeding — formula	*("infant formula"[MeSH Terms] OR "formula feeding"[Title/Abstract] OR "formula-fed"[Title/Abstract] OR "bottle feeding"[MeSH Terms] OR "mixed feeding"[Title/Abstract])*
#3	Exposure (combined)	*(#1 OR #2)*
#4	Oral microbiota — generic	*("microbiota"[MeSH Terms] OR "microbiome"[Title/Abstract] OR "oral microbiome"[Title/Abstract] OR "oral microbiota"[Title/Abstract] OR "oral microbiome"[MeSH Terms])*
#5	Oral microbiota — sequencing	*("16S rRNA"[Title/Abstract] OR "16S ribosomal RNA"[Title/Abstract] OR "metagenomics"[Title/Abstract] OR "next-generation sequencing"[Title/Abstract] OR "microbial diversity"[Title/Abstract] OR "microbial community"[Title/Abstract])*
#6	Oral microbiota — site specific	*("saliva"[MeSH Terms] OR "dental plaque"[MeSH Terms] OR "oral bacteria"[Title/Abstract] OR "salivary microbiome"[Title/Abstract] OR "plaque microbiome"[Title/Abstract])*
#7	Microbiota (combined)	*(#4 OR #5 OR #6)*
#8	Caries outcomes	*("dental caries"[MeSH Terms] OR "early childhood caries"[Title/Abstract] OR "ECC"[Title/Abstract] OR "tooth decay"[Title/Abstract] OR "dmft"[Title/Abstract] OR "dmfs"[Title/Abstract] OR "cariogenic"[Title/Abstract])*
#9	Cariogenic bacteria	*("Streptococcus mutans"[MeSH Terms] OR "Streptococcus mutans"[Title/Abstract] OR "mutans streptococci"[Title/Abstract] OR "Lactobacillus"[MeSH Terms] OR "Veillonella"[Title/Abstract] OR "dental biofilm"[Title/Abstract])*
#10	Caries (combined)	*(#8 OR #9)*
#11	Population	*("infant"[MeSH Terms] OR "infant"[Title/Abstract] OR "newborn"[Title/Abstract] OR "neonate"[Title/Abstract] OR "child, preschool"[MeSH Terms] OR "toddler"[Title/Abstract] OR "child"[MeSH Terms])*
**#12**	**Final set**	***#3 AND #7 AND #11***
**#13**	**Date filter**	***AND ("2010/01/01"[Date - Publication] : "2026/06/06"[Date - Publication])***
**#14**	**Final query**	***#12 AND #13 — Retrieved: 612 records***

2. Scopus

Line	Concept	Search string
#1	Breastfeeding / human milk	*TITLE-ABS-KEY("breastfeeding" OR "breast feeding" OR "breastfed" OR "breast milk" OR "human milk" OR "lactation" OR "colostrum")*
#2	Formula / bottle feeding	*TITLE-ABS-KEY("infant formula" OR "formula feeding" OR "formula-fed" OR "bottle feeding" OR "mixed feeding" OR "formula milk")*
#3	Exposure combined	*(#1 OR #2)*
#4	Oral microbiota / microbiome	*TITLE-ABS-KEY("oral microbiome" OR "oral microbiota" OR "salivary microbiome" OR "dental plaque microbiome" OR "oral microbial" OR "oral bacteria")*

#5	16S / sequencing	*TITLE-ABS-KEY("16S rRNA" OR "16S ribosomal" OR "metagenom*" OR "next-generation sequencing" OR "microbial diversity" OR "microbial community" OR "amplicon sequencing")*
#6	Microbiota combined	*(#4 OR #5)*
#7	Caries / ECC	*TITLE-ABS-KEY("dental caries" OR "early childhood caries" OR "ECC" OR "tooth decay" OR "dmft" OR "dmfs" OR "cariogenic" OR "Streptococcus mutans" OR "mutans streptococci")*
#8	Population	*TITLE-ABS-KEY("infant" OR "newborn" OR "neonate" OR "toddler" OR "preschool child" OR "child" OR "baby" OR "paediatric" OR "pediatric")*
**#9**	**Final query**	***#3 AND #6 AND #8 — Retrieved: 2,931 records***

3. Web of Science Core Collection

Line	Concept	Search string
#1	Breastfeeding/human milk	*TS=("breastfeeding" OR "breast feeding" OR "breastfed" OR "breast milk" OR "human milk" OR "lactation")*
#2	Formula feeding	*TS=("infant formula" OR "formula feeding" OR "formula-fed" OR "bottle feeding" OR "mixed feeding")*
#3	Oral microbiota	*TS=("oral microbiome" OR "oral microbiota" OR "salivary microbiome" OR "16S rRNA" OR "microbial diversity" OR "oral bacteria" OR "oral microbial community")*
#4	ECC / caries	*TS=("dental caries" OR "early childhood caries" OR "ECC" OR "cariogenic" OR "Streptococcus mutans" OR "dmft")*
#5	Population	*TS=("infant" OR "newborn" OR "neonate" OR "toddler" OR "preschool" OR "child")*
**#6**	**Final query**	***(#1 OR #2) AND #3 AND #5 — Timespan 2010–2026 — Retrieved: 2,358 records***

4. Embase

Line	Concept	Search string
#1	Breastfeeding (EMTREE + free text)	*'breast feeding'/exp OR 'breast milk'/exp OR 'human milk':ab,ti OR 'breastfed':ab,ti OR 'lactation':ab,ti OR 'colostrum':ab,ti*
#2	Formula feeding (EMTREE + free text)	*'infant formula'/exp OR 'bottle feeding'/exp OR 'formula feeding':ab,ti OR 'formula-fed':ab,ti OR 'mixed feeding':ab,ti*
#3	Oral microbiota (EMTREE + free text)	*'oral microbiome':ab,ti OR 'oral microbiota':ab,ti OR 'saliva microbiome':ab,ti OR '16S ribosomal RNA':ab,ti OR 'oral bacteria':ab,ti OR 'microbial diversity':ab,ti*
#4	ECC / caries (EMTREE)	*'dental caries'/exp OR 'early childhood caries':ab,ti OR 'ECC':ab,ti OR 'Streptococcus mutans'/exp OR 'cariogenic bacteria':ab,ti OR 'dmft':ab,ti*
#5	Population (EMTREE)	*'infant'/exp OR 'newborn'/exp OR 'preschool child'/exp OR 'infant':ab,ti OR 'neonate':ab,ti OR 'toddler':ab,ti*
**#6**	**Final query**	***(#1 OR #2) AND #3 AND #5 AND [2010–2026]/py — Retrieved: 2,681 records***

https://doi.org/10.3390/nu18132138

Supplementary Table S2

Data Extraction of Included Studies

Study	N (BF/FF/Mix)	Age at sampling	De-sign	Exposure definitions (BF / FF / Mixed)	Microbiological method & sample	Alpha-diversity (index & result)	Beta-diversity (index & result)	Key taxa associated with feeding	Caries outcome	Methodological notes and caveats
**Al-Shehri et al. (2016) [19]**	Total: 38; BF: 20; FF: 10; Mix: 8	4–8 weeks	Cross-sectional	BF: exclusive breastfeeding, no supplements; FF: exclusive formula feeding; Mixed: breastfeeding plus formula	16S rRNA Roche 454 sequencing, V1–V2 region; HOMD reference database; buccal swab	Shannon index and OTU richness; FF infants showed higher alpha diversity than BF infants ($p < 0.05$)	Weighted and unweighted UniFrac; significant difference between BF and FF groups ($p < 0.05$)	BF: higher Actinobacteria and Proteobacteria; FF: higher Bacteroidetes and *Prevotella*	Not measured	No confounder adjustment; self-reported feeding exposure; small sample size (n = 38)
**Holgerson et al. (2013) [20]**	Total: 207; BF: 120; FF: 50; Mix: 37	3 months	Cross-sectional	BF: exclusive breastfeeding, no formula; FF: exclusive formula feeding; Mixed: breastfeeding plus formula	HOMIM, culture and qPCR for *Lactobacillus*; saliva swab	Number of positive HOMIM taxa; FF infants showed higher richness than BF infants (8.9 vs. 7.2 taxa; $p < 0.05$)	Hierarchical clustering; oral microbial clusters differed by feeding group	BF: higher *Lactobacillus* spp. detection (27–32% vs. 0%; p = 0.006); FF: higher *Haemophilus* spp.	Not measured	Population-based cohort; prospective feeding diary; partial confounder adjustment
**Oba et al. (2020) [21]**	Total: 12; BF: 4; FF: 4; Mix: 4	0–6 months	Cross-sectional	BF: exclusive breastfeeding; FF: exclusive formula feeding; Mixed: breastfeeding plus formula	16S rRNA MiSeq sequencing, V4 region; SILVA database; QIIME2; ASV-based analysis; buccal swab	Shannon index, Faith's phylogenetic diversity and observed ASVs; FF infants showed higher diversity than BF infants ($p < 0.005$); infants receiving solid foods showed the highest diversity	Bray–Curtis dissimilarity; PERMANOVA showed significant differences by feeding group ($p < 0.005$)	BF: higher *Streptococcus* (p = 0.02); FF: higher *Actinomyces* and *Prevotella*	Not measured	Very small convenience sample; underpowered; no confounder adjustment
**Butler et al. (2022) [22]**	Total: 39; BF: 20; FF: not applicable; Mix: 19	2, 8 and 20 months	Longitudinal cohort	BF: high breastfeeding, defined as ≥80% of feeds as breastmilk; FF: not applicable as a separate group; Mixed: any formula supplementation	16S rRNA Ion Torrent sequencing, V1–V2 region; HOMD reference database; QIIME; buccal swab	Shannon index and observed OTUs; BF infants showed higher alpha diversity than mixed-fed infants at 2 months (p = 0.0016), with attenuation by 8 months	Bray–Curtis dissimilarity, UniFrac and PERMANOVA; significant difference at 2 months, with convergence by 8 months	BF at 2 months: higher *Streptococcus mitis* group (p = 0.006); Mixed: higher *Veillonella*	Not measured	Linear mixed models adjusted for neighbourhood socioeconomic environment, mode of delivery, antibiotics and solid food intake; VicGeneration cohort
**Lif Holgerson et al. (2020) [23]**	Total: 206; BF: approximately 80; FF: approximately 40; Mix:	2 days, 3 months, 18 months, 3 years and 5 years	Longitudinal cohort	BF: exclusive breastfeeding, no supplements; FF: exclusive formula feeding;	16S rRNA MiSeq sequencing, V3–V4 region; SILVA database;	Shannon index and observed OTUs; feeding-related differences were detected only at	Bray–Curtis dissimilarity; PERMANOVA with FDR correction; exclusive breastfeeding and formula feeding dif-	At 3 months only: higher Lactobacillales in BF infants; higher richness in FF infants	Not measured	Feeding-related differences only analysable at the 3-month time point; later time points underpowered for feeding-group comparisons

	approximately 40			Mixed: breastfeeding plus formula	mothur pipeline; saliva collected using Salivette	3 months and not at later time points	fered significantly at 3 months (FDR-adjusted $p < 0.001$), with mixed-fed infants showing an intermediate profile			
**Dzidic et al. (2018) [18]**	Total: 90; BF: approximately 30 with breastfeeding ≥12 months; FF: approximately 30 with breastfeeding <6 months; Mix: approximately 30 with breastfeeding 6–12 months	Oral microbiota: 3 months to 7 years; dental caries assessment: 9 years	Prospective cohort	BF: breastfeeding duration ≥12 months; FF/short breastfeeding comparator: breastfeeding duration <6 months; Mixed/intermediate duration: breastfeeding duration 6–12 months	16S rRNA MiSeq sequencing, V3–V4 region; SILVA database; QIIME; OTUs clustered at 97%; saliva swab	Shannon index, Chao1 and observed OTUs; breastfeeding ≥12 months was associated with higher diversity than breastfeeding <6 months at 24 months and 7 years ($p < 0.05$)	Bray–Curtis dissimilarity; PERMANOVA showed significant divergence by breastfeeding duration at 24 months and 7 years ($p = 0.002$)	Breastfeeding ≥12 months: higher Lactobacillales and higher microbial diversity; *Streptococcus cristatus* abundance at 3 months was associated with dental caries at 9 years ($p = 0.026$)	Clinically assessed dental caries at 9 years using dmft/dmfs; breastfeeding ≥12 months was associated with lower caries experience than breastfeeding <6 months	Key study linking early oral microbiota with later caries outcomes; prospective feeding diary; multi-adjusted longitudinal modelling; caries measured after the ECC age window, so outcome should be interpreted as later childhood dental caries rather than ECC
**Kennedy et al. (2019) [27]**	Total: 59; BF: 31 with exclusive breastfeeding ≥5 months; FF/early formula exposure: 4 with exclusive breastfeeding 0–2 months; Mix: 14 with exclusive breastfeeding 3–4 months	6, 12 and 24 months	Prospective cohort	BF: exclusive breastfeeding duration ≥5 months; FF/early formula exposure: exclusive breastfeeding 0–2 months; Mixed/intermediate exposure: exclusive breastfeeding 3–4 months	16S rRNA MiSeq sequencing, V3–V4 region, 2 × 300 cycles; mothur and VSEARCH; SILVA v128; OTUs clustered at 97%; rarefied to 10,472 reads; evening saliva	Inverse Simpson index; breastfeeding was not associated with alpha diversity; age was the main determinant, with approximately threefold increase from 6 to 24 months ($p < 0.001$)	NMDS based on Bray–Curtis dissimilarity; PERMANOVA/adonis II showed breastfeeding associated with beta diversity ($p = 0.02$) and phylum distribution ($p < 0.01$), although no specific phylum was confirmed in post-hoc testing	No specific OTU associated with breastfeeding in confirmatory sensitivity analysis; antibiotics associated with higher Pasteurellaceae and Neisseriaceae and lower Prevotellaceae	Not measured	DAG-based confounder selection; FDR 10%; ordinal logistic sensitivity analysis; first sampling at 6 months, after likely introduction of complementary foods, so early feeding effects may have been attenuated; breastfeeding categorised as ≥5 months regardless of solid food introduction
**Davis et al. (2022) [24]**	Total: 33; BF: 20; FF: 0; Mix: 13	6 weeks	Cross-sectional	BF: exclusively breastmilk-fed; FF: no formula-only group; Mixed: breastmilk plus formula	16S rRNA MiSeq sequencing, V3–V4 region; SILVA database; DADA2; ASV-based analysis; buccal swab	Shannon index and observed ASVs; mixed-fed infants showed higher Shannon diversity than exclusively breastfed infants ($p < 0.05$)	Bray–Curtis dissimilarity; PERMANOVA showed significant difference between exclusively breastfed and mixed-fed infants ($p < 0.05$)	Exclusively breastfed infants: higher *Streptococcus* and *Gemella*; Mixed-fed infants: higher *Veillonella*	Not measured	Comparator was mixed feeding rather than exclusive formula feeding; small sample size; no adjustment for neighbourhood socioeconomic environment
**Kageyama et al. (2022) [25]**	Total: 448; BF: 255; FF: 60; Mix: 131	4 months	Cross-sectional	BF: exclusive breastfeeding, no formula; FF: exclusive formula feeding; Mixed: breastfeeding plus formula	PacBio Sequel II full-length 16S rRNA sequencing, V1–V9 region; DADA2;	Observed ASVs; FF infants had higher richness than mixed-fed and BF infants: FF 43.9 ± 17.7 ASVs, Mixed	Bray–Curtis dissimilarity and PCoA; significant differences between feeding groups ($p < 0.001$); FF infants	FF: higher *Prevotella melaninogenica*, *Granulicatella adiacens* and maternally derived oral bacteria; BF: lower	Not measured	Largest included sample; full-length 16S sequencing provided higher taxonomic resolution; analyses adjusted for mode of delivery, antibi-

					eHOMD; ASV-based analysis; tongue swab	32.1 ± 13.6, BF 27.3 ± 11.3 (p < 0.001)	showed greater similarity to maternal oral microbiota	maternal bacterial transmission		otics and birthweight; Kruskal–Wallis tests with FDR correction
**Eshriqui et al. (2020) [16]**	Total: 423; BF/no formula: 175; FF/any formula: 248; Mix: included within formula-exposed group	Feeding exposure: first 6 months of life; oral microbiota: 10–14 years	Retrospective cross-sectional sensitivity study	BF/no formula: no infant formula during the first 6 months; FF/any formula: any formula use during the first 6 months; Mixed: included in formula-exposed group	16S rRNA HiSeq1500 sequencing, V3–V4 region; mothur; SILVA v119; OTUs clustered at 98%; saliva collected using Oragene OG-500	Shannon and Inverse Simpson indices; no significant difference by infant formula exposure (Shannon p = 0.877; Inverse Simpson p = 0.949)	Bray–Curtis dissimilarity; PERMANOVA showed no global difference by early formula exposure (p = 0.881)	No formula exposure: higher *Eubacterium* OTU#019 and *Veillonella* OTU#232/OTU#158; three specific OTUs differed significantly (all p < 0.001)	Not measured	Sensitivity study only: oral microbiota measured 10–14 years after feeding exposure; retrospective recall bias; substantial unmeasured intervening confounding from diet, hygiene, antibiotics, fluoride and environmental exposures; interpreted separately throughout the synthesis
**Ramadugu et al. (2021) [26]**	Total: 101; BF: 21; FF: 37; Mix: 42	2, 9/12 and 24 months	Prospective cohort	BF: exclusive breastfeeding at 2 months; FF: formula only at 2 months; Mixed: breastmilk plus formula at 2 months	16S rRNA HiSeq sequencing, V6 region, 100 bp paired-end; MED oligotyping; CORE oral microbiome database; saliva collected using DNA Genotek kit	Shannon index and Chao1; BF infants had lower Shannon diversity than FF infants at 2 months (2.77 vs. 3.12; p = 0.003), with attenuation by 12 months	Bray–Curtis dissimilarity and longitudinal mixed models; significant feeding-group differences at 2 months, with convergence by 12 months	BF at 2 months: lower *Veillonella* (−37.3%), lower *Prevotella* (−47.3%) and lower *Granulicatella* (−26.4%); maternal DMFT >0 associated with higher *Veillonella* and *Actinomyces* in infants	Not measured; infants had insufficient erupted teeth for caries assessment	Longitudinal mixed models adjusted for area, mode of delivery and maternal DMFT; qPCR for *S. mutans* and *Candida albicans* provided additional microbiological information; COHRA2 Appalachia cohort, potentially limiting generalisability; V6 region has lower taxonomic resolution
**Timby et al. (2017) [17]**	Total: 240; BF/BFR: 80; FF: 80 + 80; Mix: not applicable	4 and 12 months	Randomised controlled trial of formulas plus non-randomised breastfeeding reference group	BF: breastfeeding reference group, exclusively breastfed from enrolment before 2 months; FF: standard formula or bovine milk fat globule membrane-enriched formula; Mixed: no mixed-feeding group	16S rRNA MiSeq sequencing, V3–V4 region; HOMD; HOMINGS/Forsyth pipeline; buccal, tongue and alveolar mucosa swabs, with tooth samples at 12 months	OTU richness from rarefaction curves; breastfeeding reference group had lower richness than formula-fed groups at 4 months (p < 0.05), but the difference was not significant at 12 months	PCoA based on Bray–Curtis dissimilarity and PLS regression; breastfeeding reference group was distinct from formula-fed groups at 4 months (Q^2 = 57%) and remained partly distinct at 12 months (Q^2 = 30%)	Breastfeeding reference group at 4 months: higher *Lactobacillus gasseri*, *Lactobacillus rhamnosus*, *Bifidobacterium breve* and *Kingella oralis*; at 12 months: lower *Streptococcus mutans* prevalence than formula-fed groups	Not measured	Breastfeeding reference versus formula comparison is observational because the breastfeeding reference group was not randomised; randomised component has lower risk of bias for the formula-composition comparison, not for breastfeeding versus formula feeding; PLS Q^2 values are not directly combinable with other beta-diversity statistics

Supplementary Figure S1

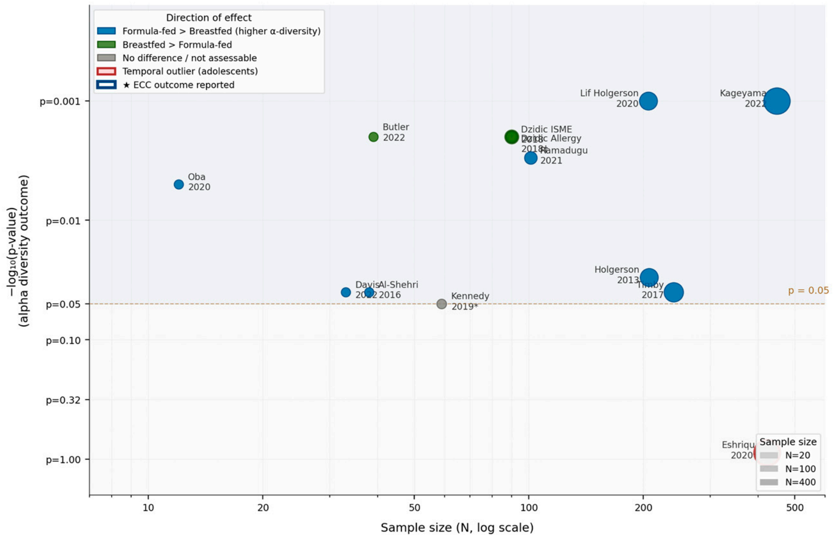

Supplementary Figure S1. *Albatross plot of included studies. Each bubble represents one study. X-axis: sample size (log scale). Y-axis: −log_{10}(p-value) for alpha-diversity outcome. Bubble area is proportional to sample size. Blue = formula-fed infants showed higher alpha-diversity; green = breastfed infants showed higher alpha-diversity; grey = no significant difference or not assessable. The dashed horizontal line indicates p = 0.05. BF, breastfed; FF, formula-fed. *Kennedy et al. (2019) [27]: p-value estimated from the abstract only. †Eshriqui et al. (2020) [16]: microbiota measured 10–14 years after feeding exposure.*

https://doi.org/10.3390/nu18132138
